# Murine Factor H Co-Produced in Yeast With Protein Disulfide Isomerase Ameliorated C3 Dysregulation in Factor H-Deficient Mice

**DOI:** 10.3389/fimmu.2021.681098

**Published:** 2021-05-12

**Authors:** Heather Kerr, Andrew P. Herbert, Elisavet Makou, Dariusz Abramczyk, Talat H. Malik, Hannah Lomax-Browne, Yi Yang, Isabel Y. Pappworth, Harriet Denton, Anna Richards, Kevin J. Marchbank, Matthew C. Pickering, Paul N. Barlow

**Affiliations:** ^1^ Centre for Inflammation Research, Queen’s Medical Research Institute, University of Edinburgh, Edinburgh, United Kingdom; ^2^ School of Chemistry, University of Edinburgh, Edinburgh, United Kingdom; ^3^ Centre for Inflammatory Disease, Imperial College London, London, United Kingdom; ^4^ Translational and Clinical Research Institute, Newcastle University, Newcastle, United Kingdom; ^5^ National Renal Complement Therapeutics Center, Royal Victoria Infirmary, Newcastle, United Kingdom; ^6^ School of Biological Sciences, University of Edinburgh, Edinburgh, United Kingdom

**Keywords:** complement system, factor H, C3 glomerulonephritis mouse model, therapeutic protein, C3 glomerulonephritis, chaperonin, *Pichia pastoris*, protein disulfide isomerase (PDI)

## Abstract

Recombinant human factor H (hFH) has potential for treating diseases linked to aberrant complement regulation including C3 glomerulopathy (C3G) and dry age-related macular degeneration. Murine FH (mFH), produced in the same host, is useful for pre-clinical investigations in mouse models of disease. An abundance of FH in plasma suggests high doses, and hence microbial production, will be needed. Previously, *Pichia pastoris* produced useful but modest quantities of hFH. Herein, a similar strategy yielded miniscule quantities of mFH. Since FH has 40 disulfide bonds, we created a *P. pastoris* strain containing a methanol-inducible codon-modified gene for protein-disulfide isomerase (PDI) and transformed this with codon-modified DNA encoding mFH under the same promoter. What had been barely detectable yields of mFH became multiple 10s of mg/L. Our PDI-overexpressing strain also boosted hFH overproduction, by about tenfold. These enhancements exceeded PDI-related production gains reported for other proteins, all of which contain fewer disulfide-stabilized domains. We optimized fermentation conditions, purified recombinant mFH, enzymatically trimmed down its (non-human) N-glycans, characterised its functions *in vitro* and administered it to mice. In FH-knockout mice, our de-glycosylated recombinant mFH had a shorter half-life and induced more anti-mFH antibodies than mouse serum-derived, natively glycosylated, mFH. Even sequential daily injections of recombinant mFH failed to restore wild-type levels of FH and C3 in mouse plasma beyond 24 hours after the first injection. Nevertheless, mFH functionality appeared to persist in the glomerular basement membrane because C3-fragment deposition here, a hallmark of C3G, remained significantly reduced throughout and beyond the ten-day dosing regimen.

## Introduction

Inheriting a deficiency in the ability to regulate the alternative pathway (AP) of complement (1, 2) predisposes to disease (3). Atypical haemolytic uraemic syndrome (aHUS) (4) and C3 glomerulopathy (C3G) (5) are kidney disorders in which symptoms arise from excessive AP activation (6). Age-related macular degeneration (AMD), a cause of blindness (7), is also linked to aberrant complement regulation (8, 9) although the mechanism is not fully established (10). In these and other complement-related diseases, such as paroxysmal nocturnal haemoglobinuria, treatments to suppress complement are in clinical use, with more in the pipeline (11, 12).

Eculizumab, a mAb to complement protein C5 (13) is approved for treating PNH (14) and aHUS (15), but showed less promise in AMD (16) and C3G (17). Anti-VEGF drugs are approved for wet AMD but dry AMD, likely to cause central-vision loss in 5-10 million individuals by 2040 (18), is harder to treat. Marginal success was reported for a PEGylated complement C3-inhibitor peptide (19), while an anti-complement factor D mAb did not meet clinical phase-II milestones (20). Other antibodies and engineered-protein therapeutics are in development (21), but there is no treatment for dry AMD, nor for C3G. An alternative is to replace deficient or defective genes or proteins. Precedence for protein replacement is provided by plasma infusions to treat complement dysregulation-linked aHUS (22–25), but regular whole-plasma infusions may fail long term (26).

Candidates for individual protein-replacement therapy include factor I (FI) (27, 28) and its co-factor factor H (FH) (29, 30). Circulating in plasma, FI and FH regulate production of the protein C3b. In the AP (1), C3 cleavage continuously generates C3b and the anaphylatoxin C3a. Nascent C3b remains in solution or binds covalently to nearby surfaces. Proenzyme factor B (FB) can bind to C3b whereupon FB is cleaved by factor D (FD) to form C3bBb. C3bBb converts more C3 molecules to C3b. Numbers of both fluid-phase and opsonic, surface-bound, C3b molecules thus grow rapidly (31) unless this is halted by regulators. Self (host) cells, unlike most bacteria, display their own membrane-bound regulators of C3b amplification. This implies FI and FH (32) are needed to suppress C3b amplification in fluid phase and on non-membrane enclosed structures, including extracellular matrix, that are recognised as “self” by FH. On self surfaces, FH accelerates decay of surface-bound C3bBb and is a cofactor for C3b proteolysis by FI to iC3b. iC3b, also an opsonin, can be cleaved to yield soluble C3c and C3dg, which is further degraded to C3d and resides long-term on the surface (3). Mutations and SNPs in the FH gene (*CFH*) pre dispose to disease (33, 34). Some are highly penetrant (35, 36). Crucially, FH has other functions (37) than suppressing complement, and the loss of one or more of such “non-canonical” roles may contribute to dry AMD (38). Administering fully functional full-length FH to patients with disease-causing loss-of-function *CFH* mutations has the advantage that it makes no assumptions about which function of FH is lost or which molecular mechanism underlies disease.

*CFH*-deficient mice, an animal model of C3G (39), allow exploration of FH-replacement therapy. Unregulated C3b amplification in *CFH* ^-/-^ mice depletes plasma C3, as seen in some C3G patients. In humans with C3G, C3 fragments are covalently deposited (40) on surfaces such as the glomerular basement membrane (GBM). Similar linear electron-dense deposits are observed along the GBM of *CFH* ^-/-^ mice. Deficiency of both FH and Crry, a mouse-specific transmembrane complement regulator, yielded milder renal injury (41). Homozygosity for mouse (m)FH mutant W1206R (*i.e.*, disease-related W1183R in human (h)FH) that lacks surface-recognition functionality, causes a more acute and aHUS-like phenotype than total FH deficiency (42). Fed a high-fat diet, aged *CFH^+/-^* mice develop ocular symptoms reminiscent of AMD (43). In trials of FH-replacement therapy in *CFH* ^-/-^ mice, serum-derived hFH restored C3 levels and attenuated glomerular iC3b deposits, but formation of anti-hFH antibodies precluded long-term studies (29). Similar outcomes were achieved in *CFH* ^-/-^ mice crossed with transgenic mice expressing human *CFH* (44). Studies using FH purified from murine plasma further supported the case for therapeutic intervention with FH (45). Given the abundance of FH in plasma (200-300 mg/L), multiple-tens of mg are required for a robust animal trial of systemic administration while kilograms of therapeutic-grade recombinant FH would be needed for clinical trials. Were intravitreal injection of small doses of recombinant FH to prove effective for AMD treatment, large quantities of affordable material would still, eventually, be needed for a global rollout.

Hence an obstacle to FH therapy is access to sufficient protein. FH (32) is a soluble 155-kDa single-chain glycoprotein. Like other regulators of complement activation (RCA) family members (46), it contains a chain of complement-control protein modules (CCPs) (aka short consensus repeats); 20 in the case of FH (47). Attempts to produce hFH using mammalian (Cos7) (48) and insect cells (49) yielded sub-mg quantities. Up to 1 mg/L of pure recombinant hFH (with partially mammalian-type N-glycans) was achieved using the moss *Physcomitrella patens* (50, 51), allowing an encouraging small, short-timescale, trial in *CFH^-/-^* mice (51). Scaling this up for clinical trials would be very challenging. Recombinant production of 10-mg quantities of FH was, eventually, achieved (52) in *Pichia pastoris* (53). After harvest of secreted FH its *P. pastoris*-specific high-mannose N-glycans were trimmed to single GlcNAc residues. As expected (54), trimmed hFH retained biological functionality *in vitro* (52). Yields of hFH from a synthetic gene far exceeded the undetectable levels obtained from a native-sequence gene (52), but were 50-fold lower than those of smaller FH segments produced in *P. pastoris* (55), and orders of magnitude lower than those of many other proteins made in this host (56).

Herein, we aimed to produce sufficient mFH for extensive trials in mouse models. Use of the *P. pastoris*-based strategy that had proved moderately successful for hFH failed, with yields lower even than those reported for moss (50, 51). To make FH, all of its 20 domains, each containing two disulfides, must fold correctly. We hypothesized that limited levels of protein disulfide isomerase (PDI) activity within the endoplasmic reticulum (ER) might retard production. To test this, we applied a PDI-co-expression-in-*P. pastoris* strategy to mFH and hFH production. This afforded production gains exceeding those reported for other categories of (generally smaller) proteins. The mFH produced was active *in vitro* as well as *in vivo* albeit with a short serum half-life. Despite this, mFH treatment reduced C3 deposits in the GBM of treated animals.

## Materials and Methods

### Synthetic Genes

An expression-optimised synthetic gene for mFH was procured from GeneArt Life Technologies (now ThermoFisher). A different version was procured from DNA 2.0 (now ATUM) (**Supplementary Figure 1A**). The GeneArt-designed DNA, supplied in pPIC3.5K, was re-cloned into pPICZαB. The DNA 2.0 gene was supplied in a proprietary *P. pastoris* vector (pJexpress 912) which (like pPICZαB) utilises zeocin selection and uses the yeast α-factor signal sequence to promote secretion of the recombinant protein. An expression-optimised gene (**Supplementary Figure 1B**) for *P. pastoris* protein disulfide isomerase (PDI) was purchased in pPIC3.5K from GeneArt Life Technologies. Maxi-preps were performed in *E. coli*, then the plasmid was linearized for integration at the *AOX1* locus with *Sac1*, phenol-chloroform extracted, and ethanol precipitated.

### Transformations

For FH production, plasmids were transformed into electrocompetent *P. pastoris* KM71H cells (Mut^S^) (Invitrogen). The pPICZαB plasmid containing GeneArt DNA for mFH was additionally transformed into X33 (Mut^+^) and SMD1168H (Mut^+^, Protease A deficient) strains. For PDI, KM71 *P. pastoris* cells (*His4^-^*) were transformed with the synthetic gene for *P. pastoris* PDI in pPIC3.5K (carrying *His4*). For all transformations, electrocompetent cells were added to linearised DNA and transferred to an electroporation cuvette for pulsing (on ice) using a Biorad Genepulser II. Immediately afterwards, ice-cold 1 M sorbitol or yeast-peptone-dextrose-sorbitol (YPDS; YPD with 1 M sorbitol) medium was added and the cuvette incubated (30°C) for three hours. For FH-producing strains, aliquots were spread on YPDS plates containing 100 µg/mL to 500 µg/mL zeocin, and incubated (30°C) until colonies grew. These were re-streaked onto fresh YPDS agar plates (containing same zeocin concentration as the original plate) and incubated for three further days (30°C). PDI-strains, after transformation, were plated onto minimal-dextrose plates that were incubated (30°C), then single colonies were re-streaked on minimal-dextrose plates and incubated for three further day (30°C).

### Small-Scale FH-Production Tests

Multiple small-scale cultures were grown from single colonies in buffered glycerol-complex medium (BMGY *i.e.*, 1% yeast extract, 2% peptone, 1% (v/v) glycerol, 400 μg/L biotin, and 0.1 M potassium phosphate, pH 6.0), before cells were transferred to 25 mL of buffered methanol-complex medium (BMMY *i.e*. 1% yeast extract, 2% peptone, 1% (v/v) methanol, 400 μg/L biotin, and 0.1 M potassium phosphate, pH 6.0). Baffled flasks containing cells suspended in BMMY were incubated at 20°C in a shaking incubator for four days with daily methanol feeds. On day 4, cells were spun out and discarded. The supernatant was filtered before addition of phenylmethylsulfonyl fluoride (PMSF) and ethylenediaminetetraacetic acid (EDTA), (to 0.5 mM and 5 mM, respectively). Protein expression was checked using sodium dodecylsulfate-polyacrylamide gel electrophoresis (SDS-PAGE) with Coomassie Blue staining or western blotting. Treatment of samples prior to SDS-PAGE with Endo H_f_, to remove *P. pastoris*-derived high-mannose N-glycans, was achieved by adding to a 50 µL aliquot of supernatant, 1 µL of a 1:3 dilution of stock enzyme (NEB, 1,000,000 units/ml) and incubating for one hour at 37°C.

### Small-Scale PDI-Production Tests

Small-scale test growths, from single colonies of *PDI*-transformed cells growing on minimal-dextrose plates, were performed as above. Both supernatants and cell pellets were retained, post-harvest, for SDS-PAGE. To obtain protein from pellets, they were vortexed with NuPAGE sample buffer containing 2% lithium dodecyl sulfate and incubated (100°C) for 120 s then re-vortexed and centrifuged. Based on the appearance of appropriate bands, a colony judged to be producing PDI (*i.e.*, KM71-PDI) was selected, and transformed with FH-encoding DNA, as above. Subsequent small-scale FH production tests were also performed as above.

### Fermentations

The best 10-L fermentation for FH production, prior to co-expression with *PDI*, was performed in a Bioflo 3000 (New Brunswick Scientific). A single colony picked after re-streaking on a 500 µg/mL-zeocin YPD plate was used to inoculate a 10-mL BMGY (500 µg/mL zeocin) seed culture that, after incubation (30°C, three days), was used to inoculate two BMGY (500 µg/mL zeocin) starter cultures in 2-L shaker flasks. After three further days at 30°C, the starter culture was inoculated into 7.5 L of fermentation basal salts medium (2.5% (v/v) glycerol, 0.425% potassium hydroxide, 1.5% magnesium sulphate heptahydrate, 1.82% potassium sulphate, 0.095% calcium sulphate, 2.3% (v/v) ortho-phosphoric acid, 0.025% (v/v) antifoam 206 (Sigma–Aldrich) and 0.4% (v/v) high-purity grade fermentation trace mineral salts (PTM1 salt, Amresco). The pH was adjusted (after inoculation) to 5.0 using 34% (v/v) ammonia solution. Cell mass was grown in a batch-mode glycerol-fed phase at 30°C (1.2 mL of PTM1 salts added for every 100 mL glycerol) to reach a wet-cell mass of 200 g/L prior to induction with 0.5% (v/v) methanol (15°C). Following induction, methanol was fed in batches so as to maintain a level of 1.5% (v/v) (1.2 ml PTM1 salts added for every 100 mL of methanol; 1% sorbitol (w/v) and 0.3% tryptone included in each methanol feed).

Fermentations of the *CFH/PDI*-co-expressing strain were performed in a similar manner except a continuous feeding process was used during induction. In this phase, a pump was set to feed methanol/glycerol (in a 4:1 mixture, contained 10 mM PMSF) at an initial 50 mL/hour, which increased daily to 88 mL/hour over a four-day induction period. Additions to achieve a final concentration of 0.3% tryptone were made approximately every four hours.

### Purification of Recombinant Murine FH

After spinning out cells, supernatant was diluted, and pH adjusted to 6.0. Factor H was captured on Toyopearl SP-650 resin (Tosoh Biosciences) (eluted with 50-500 mM NaCl gradient). After incubation with Endo H_f_ (New England BioLabs), released sugars and non-deglycosylated material were removed by passage through concanavalin A resin, then samples were dialysed into glycine buffer, pH 9.5 (90 mM NaCl). This material was loaded onto a column containing MonoQ or SourceQ (Sigma) and eluted with a gradient to 1 M NaCl. After dialysis into PBS, pH 7.4, FH was further purified by size-exclusion chromatography on Superdex 200 (Sigma). In an attempt to enrich non-“clipped” intact protein, a small aliquot from the peak fraction eluted from the size-exclusion column was loaded onto a ceramic hydroxyapatite column in 10 mM sodium phosphate, 200 mM NaCl, pH 6.5, and eluted with a gradient to 500 mM sodium phosphate, 200 mM NaCl, pH 6.5. This sample was used for co-factor assays.

### Surface Plasmon Resonance Measurements

All surface-plasmon resonance (SPR) experiments were performed on a BIAcore T200 instrument (GE Healthcare) at 25°C using HBSP^+^ (10 mM HEPES, 150 mM NaCl and 0.05% w/v surfactant P20, GE Healthcare) without MgCl_2_ unless stated. Human C3b, C3d, FD and FB were purchased from CompTech, Inc. Mouse C3b, prepared from serum-derived C3, and mouse FB also purified from serum using standard procedures, were gifts from Professor Santiago Rodriguez de Cordoba (Centro de Investigaciones Biologicas, Madrid). Murine C3d was recombinant in origin, and was a gift from Dr Jonathan Hannan (University of Colorado).

We measured in various combinations the affinities of recombinant mFH, serum-purified native mFH and hFH, for immobilized mouse and human C3b and C3d. A two-fold dilution series of FH solutions (from 2 µM to 6 nM) was injected (twice, *i.e.*, two replicates) over the chip at 50 µl/min for 180 s, followed by a dissociation-observe time of 300 s. The chip surface was regenerated between injections with a double injection of 1 M NaCl (50 µl/min for 30 s). Equilibrium data were analysed using the Biacore evaluation software and a 1:1 binding model.

We used SPR to measure the ability of FH to accelerate decay of a C3 convertase (C3bBb). A CM5 chip (GE Healthcare) was used with a running buffer of HBSP^+^ containing 1 mM MgCl_2_. A total of 1500 RU of murine C3b was amine coupled to each of flow-channels 2 and 4 (flow-channels 1 and 3 were left C3b-free and used for reference). To assemble C3 convertase in each flow-channel, a mixture of (either human or murine) 500 nM factor B and 50 nM human factor D was flowed (10 µl/minute) over the chip for 180 s. An initial dissociation phase (240 s) allowed observation of some intrinsic convertase decay (Bb loss) prior to injection with either 40 nM or 100 nM (used to measure decay of human C3 convertase by mouse FH) solutions of FH (10 µl/min for 180 s). After a further dissociation phase of 240 s, the surface was regenerated with a 30-second injection (10 µl/min) of 1 M NaCl.

### Co-Factor Assay

A standard fluid-phase co-factor assay was used to estimate *in vitro* co-factor activity of mFH for human FI-mediated cleavage of human C3b. In brief, each reaction was performed in a final volume of 20 µl that included 850 nM C3b and either 110 nM or 220 nM FI. The reaction (37°C for two hours) was started with the addition of either 300 nM or 600 nM FH (or 0 nM FH in the negative control). It was terminated by addition of NuPAGE lithium dodecyl sulfate sample buffer and NuPAGE reducing agent, and then heated at 100°C for 120 s. The presence of products (*i.e.* cleaved α’-chain) was detected by the appearance of bands in SDS-PAGE.

### *In Vivo* Studies With Recombinant FH

*CFH* ^-/-^ mice were generated as previously described (39) and backcrossed onto the C57BL/6 genetic background. Mice used were 7-9 weeks old. All experimental procedures were conducted in accordance with institutional guidelines. Blood was collected into tubes containing 10 mM potassium EDTA in distilled water, *via* tail venesection, before injection and at indicated time points thereafter. Plasma was separated by refrigerated centrifugation (2000 g for 600 s) prior to storage at -80°C. Mice were culled at indicated time points, and kidneys collected into PBS and snap frozen in optimal cutting temperature (OCT) embedding matrix (CellPath) for storage at -80°C. All proteins administered to the animals were subjected to lipopolysaccharide (LPS) removal (57) and were confirmed to be LPS-free by the method of Moesby et al. (58). Protein solutions (0.5 mL in PBS vehicle) were administered by intraperitoneal (IP) injection.

For experiment 1, four mice were injected with 0.5 mg recombinant mFH, four mice received 1.0 mg recombinant mFH, two mice received serum-derived mFH and two mice received vehicle only (PBS). Mice were bled pre-injection, and at six- and 24-hours post-injection, and culled at 24 hours. For experiment 2, five mice were injected daily with 0.5 mg recombinant mFH, two mice were injected daily with 0.4 mg recombinant hFH (dictated by availability of protein at that time) and five mice were injected daily with vehicle only. Daily injections were administered for ten days in total. Mice were bled pre-injection, and at 24 hours, five days and 11 days *(i.e.*, 24 hours after final injection). Mice were culled at 11 days post-initial injection.

For the experiment to assess the immunogenicity of FH, mice (eight weeks old, wild-type and *CFH* ^-/-^) received 20 µg of protein in sterile saline *via* ip injection on Day 0 and on Day 28. Animals were bled *via* tail vein venesection into 10 mM EDTA in PBS every seven days until day 41 when they were terminally exsanguinated under anaesthesia, in accordance with animal license P35D9C60C granted by the UK Home Office.

### Measurement of C3 and FH

Levels of FH in mouse plasma were measured by capture with a sheep anti-hFH cross-reacting antibody (1:1000) (ABIN 113017) followed by detection with a cross-reacting goat anti-hFH biotinylated antibody (5 mg/ml stock) (Quidel A312) and a streptavidin alkaline phosphatase (1:2000) (BD Pharmingen 554065) reporter. Results were quantified by reference to a standard curve of recombinant murine FH protein. Levels of human FH in mouse plasma were measured by capture with an anti-FH monoclonal antibody (2 µg/ml) (Ox24, Thermo MA170057) and detection with a polyclonal sheep anti-human FH antibody (1 mg/ml) (Abcam AB8842) and a donkey anti-sheep antibody (40 µg/ml) (peroxidase-conjugated) (Jackson Lab: 713-035-147) and tetramethylbenzidine. Results were quantified by reference to a standard curve of recombinant human FH protein. To quantify production of recombinant hFH with and without PDI, an ELISA, referenced to a standard curve of human FH supplied by Complement Technologies, was used. For this assay, the N-terminal domain of PspC (prepared according to (59) was used to capture FH, and then biotinylated Ox 24 (Lifespan Biosciences), streptavidin peroxidase polymer, ultrasensitive (Sigma) and tetramethylbenzidine were used for detection. C3 levels were measured by ELISA using goat anti-mouse C3 antibody (0.5 µg/ml) (MP Biomedicals 55463) as previously described (60). Results were quantified by reference to a standard curve generated from acute-phase sera containing a known quantity of C3 (Calbiochem).

### Detection of Murine Anti-FH Antibodies in Mouse Plasma

Two ELISA protocols were used to detect anti-FH antibodies. (i) In experiments aimed at restoring complement regulation with various regimens of FH administration, MaxiSorp ELISA plates were coated with 0.0625 µg of recombinant mFH or hFH that had been diluted in 50 mM sodium carbonate, pH 9.6. After washing and blocking with 1% BSA in PBS, the plasma samples to be tested were added at a dilution of 1:50 and incubated at room temperature (one hour). After washing, plates were incubated for a further hour at room temperature with a goat anti-murine IgG Ab labelled with alkaline phosphatase (Sigma A3438). After additional washing, enzymatic activity was revealed using SIGMA-*FAST p*-nitrophenylphosphate tablets. Antibody titres were expressed as arbitrary ELISA units calculated as optical density (405 nm) of the sample multiplied by 50. (ii) In experiments designed to test immunogenicity of FH, Nunc maxiSorp ELISA plates were coated with 5 µg/ml (50 µl per well) of native or recombinant mFH diluted in PBS. After washing and blocking with 0.1% Tween in PBS, plasma to be tested was added at a dilution of 1:100 in triplicate wells and incubated (one hour, room temperature). After washing, plates were incubated for a further hour (room temperature) with sheep anti-murine IgG antibody labelled with horseradish peroxidase (anti-sera was used at a 1/2000 dilution after reconstitution in 50% v/v glycerol according to manufacturer’s instructions, #515-035-071 Jackson ImmunoResearch *via* Stratech Scientific, UK). After additional washing, enzymatic activity was revealed by addition of TMB solution (Universal Biologicals Ltd). The assay was stopped using an equal volume of 10% H_2_S0_4_, then A^450 nm^ measured. Means of triplicate readings are shown. For a positive control, a mouse anti-FH mAb (2a5, 5 mg/ml stock, a gift from Professor Claire Harris), was diluted 1/250,000 or 1/750,000. For a negative control, PBS was used. To compare the immune-reactivity of mouse terminal bleed sera generated with antigens of different origin, an ELISA protocol was performed as above, with native or recombinant mFH coated to the plate.

For Western blot (WB)-based analysis of plasma C3, samples were separated using a 10% (w/v) polyacrylamide gel under reducing conditions. The detection antibody was goat anti-serum to mouse C3 (0.8 µg/ml) (MP Biomedicals 55444) and the secondary antibody was HRP-conjugated anti-goat IgG (5.2 µg/ml) (Sigma A9452). Blots were visualized using Pierce electrochemiluminescence Western blotting substrate (Thermo Scientific). For glomerular immunostaining, cryosections from snap-frozen kidneys were fixed in acetone for 600 s. C3 was detected with FITC-conjugated anti-mouse C3 antibody (10 µg/ml) (MP Biomedicals 55500). C3d was detected using the biotin-blocking system (Dako) and a biotinylated goat anti-mouse C3d antibody (5 µg/ml) (R+D systems BAF2655) followed by Avidin-Alexa 488 (5 µg/ml) (Invitrogen S11223). IgG was visualized with a FITC-conjugated goat anti-mouse IgG antibody (11.5 µg/ml) (Sigma F5387). Twenty glomeruli were visualized per section and fluorescence intensity expressed in arbitrary units.

### Statistical Analysis

Statistical analysis was performed using GraphPad Prism (version 8.0; GraphPad). Kruskal-Wallis with Dunn’s multiple comparisons test was used when comparing multiple groups.

## Results

### Murine FH Cannot Be Made in Standard *P. pastoris* Strains

Our initial efforts to produce mFH employed a strategy used to make its human orthologue (52). Thus, we expressed in *P. pastoris* a codon-modified gene under the *AOX1* promoter. From 25-mL protein-production trials we hoped for Coomassie Blue (CB)-detectable SDS-PAGE bands verifiable by WB and, due to the presence of 40 disulfide bonds, migrating differently under reducing and non-reducing conditions. We tested *P. pastoris* KM71H, SMD1168H and X-33 (Invitrogen/ThermoFisher) transformed with plasmids containing either of two codon-modified genes for mFH. To export mFH, we explored use of mammalian and yeast secretion signals.

In the most promising trial, KM71H was transformed with a plasmid (pJe912) containing a mFH gene synthesized by DNA2.0 (now ATUM). This utilises zeocin for selection and the yeast α-mating factor for secretion. Secreted protein was Endo H_f_-treated to prune back high-mannose N-glycans. Production of mFH was so low that to obtain WB-detectable gel bands (**Supplementary Figure 2A**) we first had to enrich mFH on heparin-affinity resin. We subsequently grew this strain in a fermentor, varying zeocin concentrations and exploring growth media, feeding protocols and protease inhibitors. Most successful was a fermentation in basal-salts medium with sorbitol and tryptone added at each methanol feed over three days of post-induction culture. After cell removal, harvested supernatant was loaded on SP-sepharose. Salt elution and Endo H_f_ treatment yielded CB-detectable SDS-PAGE FH bands (**Supplementary Figures 2B, C**). Under reducing conditions only, additional protein bands appeared beneath the main band. N-terminal sequencing confirmed clipping between Arg906 and Asp907, in an eight-residue insertion with respect to hFH within CCP 15, generating two disulfide-linked polypeptides. Passage over lectin-affinity resin removed non-deglycosylated material but this, followed by anion-exchange and size-exclusion chromatography, failed to resolve clipped from non-clipped mFH. Some enrichment of non-clipped material was subsequently obtained by passage over ceramic hydroxyapatite (not shown) but it was not sufficient to justify the accompanying loss of yield.

Despite trying to optimize fermentation, yields of <1 mg/L purified mFH, were <5% of those for hFH. Efforts to reduce proteolysis, including use of protease-deficient *P. pastoris* strain SMD1168H, did not boost production or reduce clipping. Attempts to enhance gene-copy number by selecting for resistance to higher zeocin levels failed. The 40 disulfides within FH might overwhelm disulfide-forming and protein-folding machinery within *P. pastoris* ER. Thus, we explored co-expression of the synthetic mFH gene with an extraneous gene for *P. pastoris* PDI.

### Co-Expression With PDI Increases Yield > 100-Fold

To make a PDI-overproducing strain, codon-modified DNA encoding *P. pastoris* PDI was inserted into pPIC3.5K (ThermoFisher) under the *AOX1* promoter and transformed into KM71 cells to create *P. pastoris* KM71-PDI. KM71 cells have a *His4* mutation that is complemented by pPIC3.5K. KM71-PDI cells were transformed with pJe912 containing codon-modified mFH DNA, and colonies of KM71-PDI-FH selected with zeocin. Small-scale trials of the *PDI* and *mFH* co-expressing cells made sufficient mFH for WB identification without pre-enrichment, a major increase in yield over non-PDI strains (**Supplementary Figure 2D**). Using qPCR (**Supplementary Figure 3A,B**) we found two copies of the FH gene and two copies of the PDI gene were responsible for these good yields. Moreover, qRT-PCR (**Supplementary Figure 3B**) suggested that post-induction, levels of RNA transcripts for *mFH* and *PDI* were 100-fold and 200-fold higher, respectively, than those for the housekeeping (61) *β*-actin gene *Act1*.

**Figure 1:**
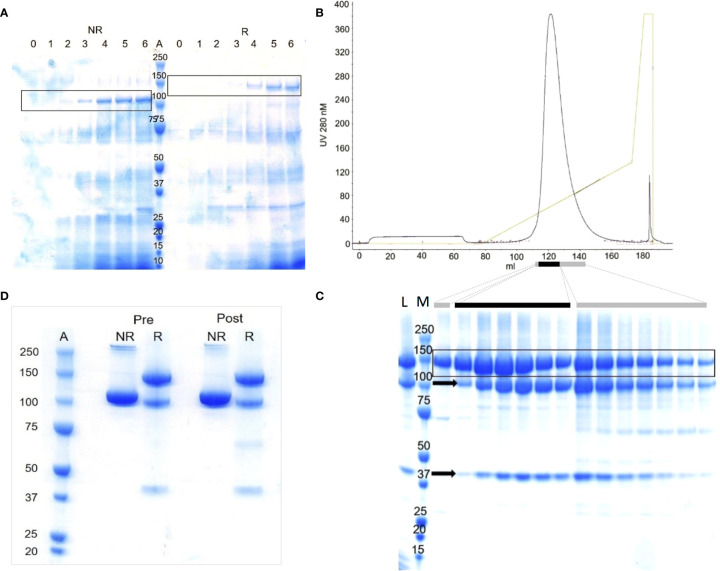
Production of recombinant mFH by fermentation of KM71-PDI-CFH.** (A)** Lanes 0-5, deglycosylated samples (10 µL) from fermentation days (d) 0-5: induced with methanol, d1; fed with methanol, d2-4; harvested, d5. Lane 6, as lane 5 with PMSF added post-harvest. Lane A, MWt markers (MWM) (kDa). NR/R; run under non-reducing/reducing conditions. **(B)** Purification on Source Q of one third of the material harvested and initially captured on cation-exchange resin; elution profile (0-1 M NaCl over 12 column volumes). **(C)** Lane L, loading sample (10 µL); Lane M, MWM; other lanes, aliquots of 2 µL (black bar) or 10 µL (grey bars) from main-peak fractions; Arrow, “clipped” protein fragments. In frames **(A, C)**, candidate rmFH bands are boxed.** (D)** Samples of recombinant mFH used *in vivo*; 5 µL loaded per well. “Pre”, before LPS removal. “Post”, after LPS removal. Lane A, MWM.

Aiming to make more mFH, exploratory ten-litre fermentations of KM71-PDI-CFH were performed. The best involved daily pumped feeds, for four days post-induction, with 4:1 glycerol:methanol plus tryptone, and daily addition of Pefabloc SC. Sampling by SDS-PAGE of the medium during induction, without concentration or enrichment, produced CB-stained candidate-mFH bands (**Figure 1A**). After centrifugation, mFH was captured from diluted supernatant by cation-exchange chromatography (Toyopearl SP-650). Eluted fractions contained predominantly mFH (**Supplementary Figures 2E, F**) but this co-purified with “clipped” mFH as before. The peak 5-mL fraction contained 4.5 mg/mL protein and pooled fractions contained 420 mg in total. These were Endo H_f_-treated, run over a lectin-affinity column, then divided into three for anion-exchange chromatography (**Figures 1B, C**), yielding 78 mg mFH in total. This exceeds by >200-fold the yield from non-*PDI* strains. Contamination with clipped mFH could not be resolved under conditions compatible with structural integrity and preservation of yield.

**Figure 2:**
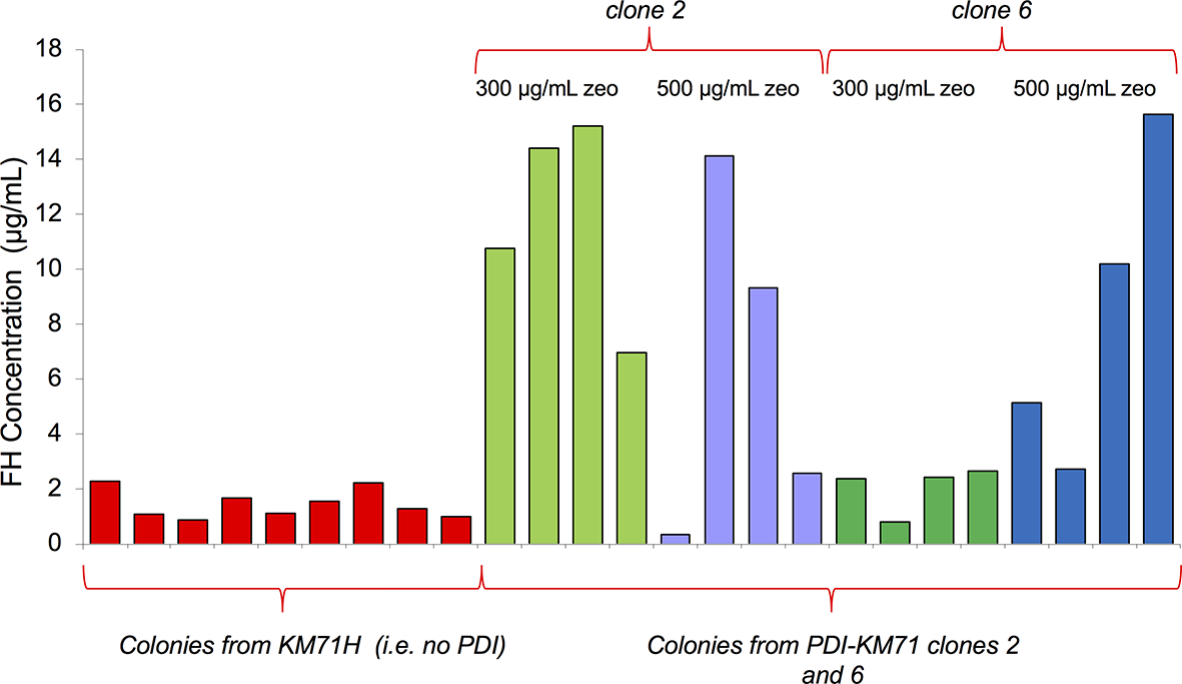
Comparison of recombinant hFH production with and without co-production of PDI. KM71H, or two colonies of KM71-*PDI*, were transformed with pPicZα containing a gene for human FH. After selection with 300 or 500 µg/mL zeocin, four single colonies in each case were used to inoculate 25-mL test growths. An ELISA (see Methods) was used to estimate recombinant hFH production in supernatants.

The yield of mFH from these *P. pastoris* cells was a striking improvement compared to cells not producing extra PDI. Unlike in the case of mFH, a codon-modified gene for hFH did express reasonably well in *P. pastoris* KM71H without extra PDI. We wondered whether co-expression with *PDI* might also improve hFH production. We transformed KM71-PDI and KM71H strains with pPicZα containing the same codon-modified FH gene used previously (52), then performed an ELISA to compare hFH production between the two strains. We found (**Figure 2**) that co-production with PDI in *P. pastoris* substantially increases hFH yields.

### *In Vitro* Tests of mFH Function

**Figure 3:**
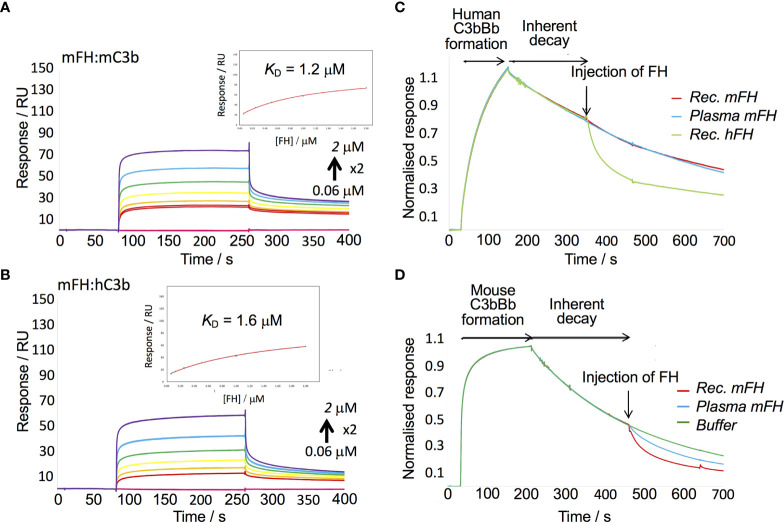
*In vitro* assays of recombinant mFH. **(A)** A recombinant mFH dilution series was flowed over murine C3b amine-coupled to a C1 chip [233 response units (RU)] in the SPR instrument, and equilibrium data plotted (insert). **(B)** The recombinant mFH was flowed over human C3b (258 RU), as in **(A)** *K*_D_ estimates in **(A, B)** assumed reversible 1:1 binding. **(C)** In a decay-acceleration assay, human C3b was coupled to a CM5 chip (1140 RU) then human FB and FD were flowed over it, forming human C3bBb. Its decay rate accelerated upon injection (arrow) of 100 nm hFH. No such increase was elicited by recombinant mFH, or FH from mouse serum. **(D)** Murine C3b was amine coupled to the CM5 chip (1510 RUs), then mouse FB and FD were injected, forming mouse C3bBb. Its decay rate was increased following injection of 40 nM mFH.

Previous work showed hFH binds human C3b, the activated fragment of C3, with a *K*_D_ of 0.3 -1.3 µM (62–64) when C3b is immobilised on an SPR chip. Binding to C3b is a prerequisite of both the cofactor activity of FH and its decay-accelerating activity (DAA) towards the C3 convertase, C3bBb (31). We therefore measured binding of our mFH to mouse C3b amine coupled to a C1 chip. By flowing recombinant mFH over C3b in the SPR instrument, and fitting responses to a 1:1 equilibrium model, we obtained a *K*_D_ of 1.2 µM, similar to that of the hFH:C3b interaction. Parallel measurements of recombinant mFH binding to human C3b yielded *K*_D_ = 1.6 µM (**Figures 3A, B**).

To measure DAA, we modified a published protocol (65). Thus, we formed mouse C3bBb by flowing FD mixed with murine FB (mFB) and murine C3 (**Figure 3D**) over murine C3b “seeds” amine coupled to a CM5 SPR chip. Subsequently, murine C3bBb irreversibly decays over several minutes. By flowing recombinant mFH over mouse C3bBb we observed decay acceleration. We found that native mFH, purified from mouse serum, was slightly less active than recombinant mFH in this assay. Minor differences between native FH and FH were likewise reported in DAA assays on hFH (52). In a parallel experiment, we found that mFH did not accelerate decay of the human C3bBb complex (**Figure 3C**). We found that complexes did not form between human C3b and mFB, or mouse C3b and human FB. We also showed that recombinant mFH had cofactor activity for cleavage of human C3b by human FI (**Supplementary Figure 4**).

### *In Vivo* Tests of Recombinant mFH Efficacy

**Figure 4:**
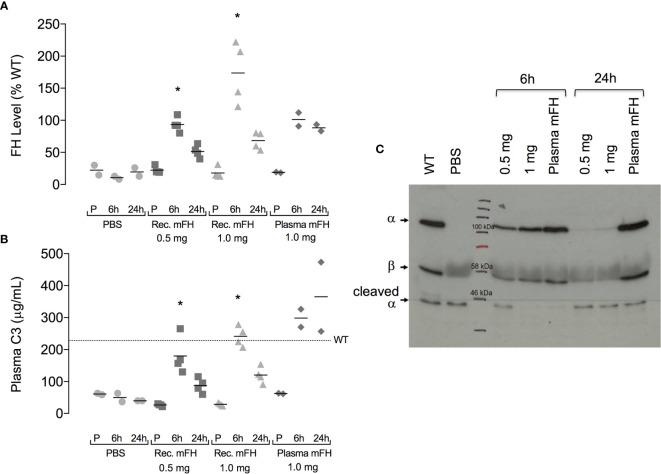
Plasma complement profile in *CFH* ^-/-^ mice after injection of mFH. **(A)** Plasma FH levels, and **(B)** plasma C3 levels, pre-injection (P) and six hours and 24 hours after IP injection of recombinant mFH (rec. mFH), FH purified from mouse serum (plasma mFH), or vehicle (PBS). Mean wild-type (WT) plasma C3 concentration (dotted line) = 230 µg/mL. Horizontal bars denote mean values. *P=0.0034 versus pre-injection value, Dunn’s Multiple Comparisons. **(C)** Western blot (reducing conditions) showing non-cleaved C3 α-chain after mFH injection.

Previously, IP injection of native (serum-derived) mFH or hFH into *CFH* ^-/-^ mice temporarily restored serum C3 levels by establishing fluid-phase regulation of C3 consumption (29, 45). It also reduced C3-fragment deposition along the GBM. We asked whether FH produced in yeast could achieve these therapeutically desirable goals. Endo H_f_-treated samples of recombinant FH were depleted of lipopolysaccharides (LPS), then dialysed into PBS and adjusted to 1-2 mg/mL protein (LPS levels < 0.1 x 10^-4^ µg/mL) ready for injection. SDS-PAGE (**Figure 1D**) confirmed that the small proportion of proteolytically clipped FH remained unchanged during these procedures. We injected, IP, *CFH* ^-/-^ mice with 0.5 mg or 1.0 mg recombinant mFH. We compared these “low-dose” and “high-dose” groups with a third group; two of these received 0.5 mg native mFH from mouse serum, and two received PBS only. Levels of FH and C3 in plasma were monitored (**Figures 4A, B**).

At six hours post-injection (p.i.), plasma FH levels in *CFH* ^-/-^ mice receiving mFH attained approximate parity with levels in non-treated wild-type (WT) mice. Plasma FH levels were more elevated for the higher than the lower dose of recombinant mFH (**Figure 4A**). At 24 hours p.i., plasma mFH had approximately halved for both doses of recombinant mFH. Conversely, plasma FH levels in *CFH* ^-/-^ mice 24 hours p.i. of native mFH remained similarly elevated to levels recorded at six hours. Hence, recombinant mFH was cleared faster than native mFH.

We measured secondary consequences of elevated plasma FH in *CFH* ^-/-^ mice by monitoring C3 (**Figure 4B**). At six hours p.i., all mice dosed with mFH had elevated plasma C3 levels suggesting restoration of control of fluid-phase activation (that otherwise depletes plasma C3). Higher C3 levels were measured in mice that received 1.0 mg recombinant mFH, or 0.5 mg of native mFH, compared to those receiving 0.5 mg recombinant mFH. These higher values resemble serum C3 levels in non-treated WT mice. At 24 hours p.i., C3 levels, like FH levels, had declined in both groups of mice receiving recombinant mFH. Conversely, plasma C3 levels did not fall at 24 hours in mice receiving native mFH. Hence C3 concentrations correlated broadly with plasma FH levels. We ascertained whether the C3 detected was intact (**Figure 4C**). Plasma C3 sampled at six hours p.i. with 1.0 mg recombinant mFH or 0.5 mg native mFH, retains an intact α-chain. After 24 hours, C3 in mice treated with native mFH remains largely intact. Conversely the lower quantities of C3 in mice receiving recombinant mFH do not possess an intact α-chain.

**Figure 5:**
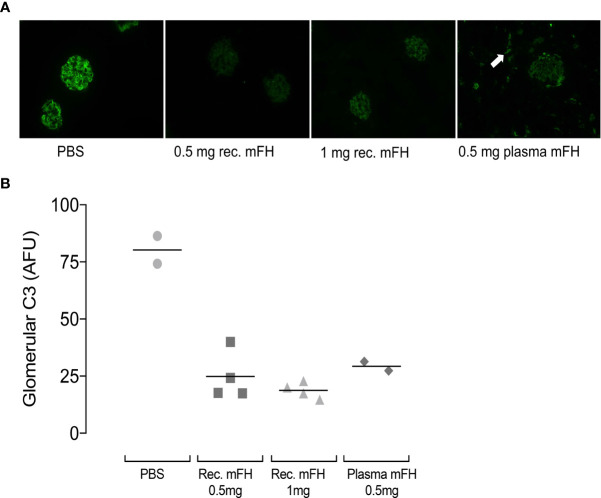
Glomerular C3 immunostaining in *CFH* ^-/-^ mice after mFH injection. **(A)** Representative images of C3 staining 24 hours after injection of FH (or vehicle) as indicated below each image. White arrow indicates tubulo-interstitial C3 staining in animals receiving mouse serum-derived FH. **(B)** Quantitation of fluorescence intensities expressed in arbitrary fluorescent units with horizontal bars denoting mean values. Rec. mFH, recombinant mFH.

Dense glomerular deposits of C3 fragments feature in C3G. We sought these in *CFH* ^-/-^ mice using C3 immunostaining. **Figure 5** shows representative comparisons of glomerular C3-fragment deposition at 24 hours p.i. for recombinant mFH, native mFH or PBS. In PBS-treated *CFH* ^-/-^ mice, linear staining of capillary walls resembles C3G (29, 39, 45). Significant reductions in capillary-wall C3 immunostaining were found in *CFH* ^-/-^ mice dosed with recombinant mFH. In *CFH* ^-/-^ mice receiving native mFH, capillary wall staining was also reduced (**Figure 5B**), and tubulo-interstitial staining evident, as reported in healthy wild-type mice (45). C3 staining was reduced 24 hours after recombinant mFH injection, despite the decline of plasma FH and C3 levels. We sought IgG staining at the GBM, indicating immune complex formation as might be triggered by anti-FH antibodies, but none was observed.

In sum, these studies show: (i) high plasma levels of mFH and intact C3 persist 24 hours after dosing *CFH* ^-/-^ mice with native mFH; (ii) dosing with recombinant mFH temporarily raised plasma mFH levels but recombinant mFH was cleared faster than native mFH; (iii) due to faster clearance, injected recombinant mFH controlled fluid-phase C3 consumption less effectively than did native mFH; (iv), the protective effects of recombinant mFH, in terms of preventing C3 fragment deposition along the GBM, persisted after serum levels of the administered recombinant protein had fallen to 50% of WT levels.

**Figure 6:**
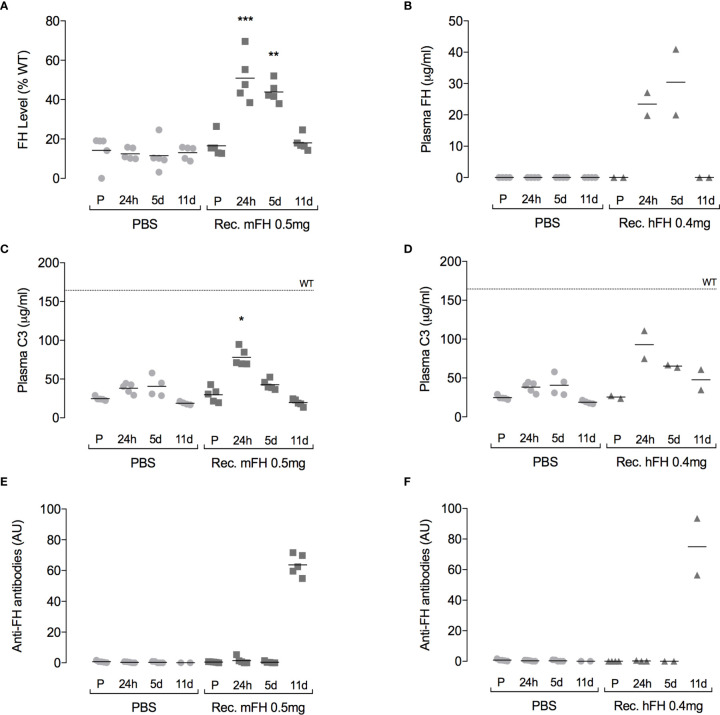
Plasma complement profile following daily FH doses. Recombinant versions of **(A, C, E)** murine FH, or **(B, D, F)** human FH were administered IP daily to *CFH^-/-^* mice for ten days. Plots show FH **(A, B)**, C3 **(C, D)** and anti-FH antibody **(E, F)** levels in plasma, pre-injection (P), and at 24 hours, five days, and 11 days after the first injection. The dotted line (at 165 µg/mL) is mean plasma C3 concentration in wild-type mice, *P=0.0192, **P=0.0255, ***P=0.0033 versus pre-injection values, Dunn’s Multiple Comparisons. Horizontal bars denote mean values.

Clearance of recombinant mFH complicates assessment of potential benefits. We therefore studied in *CFH* ^-/-^ mice the cumulative outcome of ten daily 0.5-mg IP injections of recombinant mFH. A second group of C*FH* ^-/-^ mice received PBS. In parallel, a similar dosing regimen was followed (0.4 mg daily) using recombinant hFH. Samples were taken pre-injection and at 24 hours, five days, and 11 days after the first injection of the series. At 24 hours (**Figures 6A, B**), plasma FH levels in mice receiving recombinant mFH or hFH were elevated, consistent with levels observed in the previous experiment following a single injection of recombinant mFH. Similar levels were detected at day 5, but by day 11 FH levels reverted to pre-injection values. At 24 hours (**Figures 6C, D**), levels of intact C3 in C*FH* ^-/-^ mice dosed with recombinant mFH or hFH, at ~80 µg/mL, resembled those in the previous, single-dose experiment, *i.e.*, approximately double those in mice receiving PBS. Subsequently, plasma C3 levels declined, and they did not differ significantly from PBS-injected mice at 11 days.

**Figure 7:**
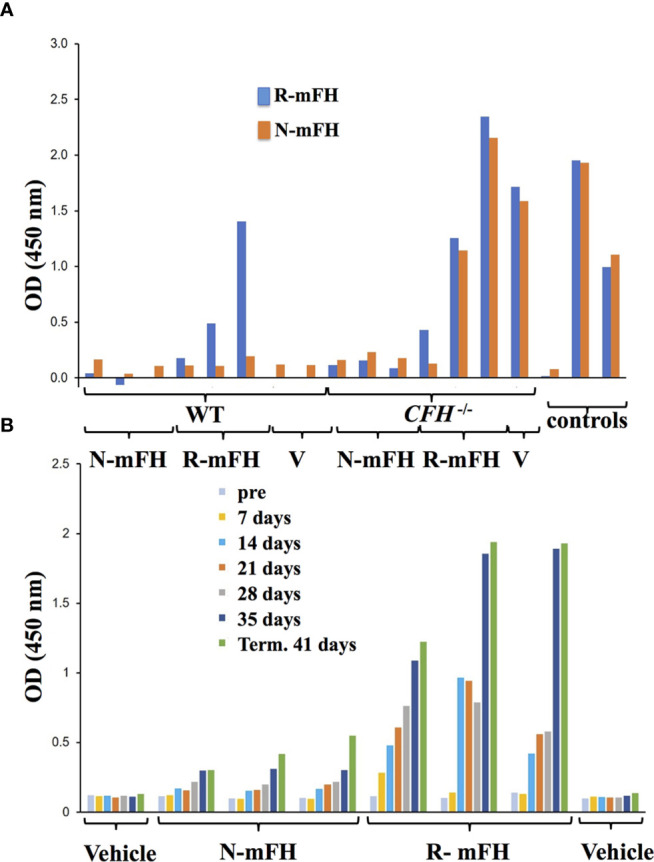
Anti-FH antibodies induced by dosing with recombinant FH. **(A)** A test of whether injections of native [plasma-derived) mFH or rmFH or vehicle (V)] into wild-type mice induce antibodies recognising native mFH (N-mFH), or recombinant mFH (R-mFH). Positive control: 1/250,000 dilution of mouse monoclonal antibody to mFH. **(B)** Emergence of anti-FH antibodies over time after dosing in three two-month-old CFH^-/-^ mice (displaying mild or little clinical phenotype at this point). OD^450 nm^ in **(A, B)** are read-outs of tetramethylbenzidine exposed to bound HRP-conjugated sheep anti-mouse IgG antibody (Jackson ImmunoResearch); the means of triplicates are shown.

We tried to detect anti-FH antibodies in *CFH* ^-/-^ mice plasma after injection of recombinant FH (**Figures 6E, F**). No anti-FH antibodies were detected at 24-hour and five-day time points, but substantial levels were evident at day 11. In a previous study of native human FH administration to *CFH* ^-/-^ mice, anti-FH antibodies were also observed after ten daily doses (29). To further investigate immunogenicity of recombinant mFH, experiments were performed in which wild-type mice and *CFH* ^-/-^ mice were injected with either recombinant mFH or native mFH (at day 0 and again at day 28), and anti-FH antibodies measured in blood weekly prior to terminal bleed at 41 days (**Figure 7**). Recombinant mFH was consistently more immunogenic than native mFH in both wild-type (**Figure 7A**) and *CFH* ^-/-^ mice (**Figure 7B**). A response to recombinant mFH is evident in wildtype mice which does not cross-react significantly with native FH (**Figure 7A**), suggesting antigenic epitopes exist on recombinant mFH that are distinct to the native FH in these mice.

**Figure 8:**
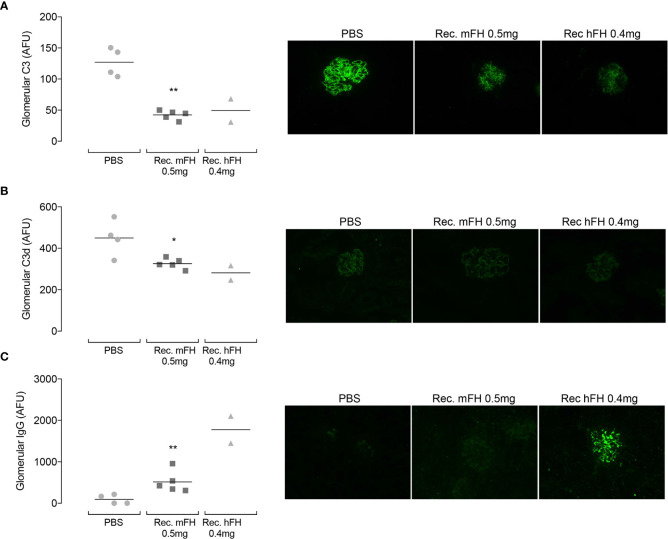
Glomerular C3 immunostaining in *CFH^-/-^* mice following repeat dosing of FH. Animals received ten daily injections of recombinant mFH or recombinant hFH (or vehicle) and were culled at day 11. Glomerular C3 **(A)**, C3d **(B)** and IgG **(C)** were assessed, and representative images shown. Quantitation of fluorescence intensity, in arbitrary fluorescent units, is plotted with horizontal bars denoting mean values. *P=0.0317, **P=0.0159 *versus* PBS, Mann-Whitney Test.

Returning to the daily-dose experiment, **Figure 8A** shows representative images of glomerular C3 immunostaining at day 11 (24 hours after tenth injection). In vehicle-only controls, linear capillary wall staining with an anti-C3 antibody is evident as expected (39). Strikingly, in mice injected with recombinant mFH or hFH, capillary wall staining is markedly reduced. This is despite plasma FH and C3, in mice receiving recombinant FH, having reverted to vehicle-only control levels (see above). **Figure 8B** shows glomerular C3d(g) immunostaining at day 11 (this antibody does not recognise C3, iC3b, C3c or C3b). C3d(g) is the product of C3b cleavage by FI and cofactors including FH. Vehicle-only control *CFH* ^-/-^ mice showed evidence of glomerular reactivity with anti-C3d(g) antibody, but reduced staining is evident in mice dosed with recombinant mFH and hFH, in agreement with a previous ten-day study (29) with native hFH in which C3d(g) staining decreased at day five and continued to decline until day 11. **Figure 8C** summarises the results of glomerular IgG immunostaining at day 11. In the previous native-hFH study (29), strong glomerular FH staining was seen at day 11 in a pattern equivalent to that seen for C3 fragments. Therefore, any IgG detected in the current study likely represents mouse IgG : FH immune complex deposition. No IgG staining is observed in vehicle-only mice, but florid IgG staining occurs in mice that received recombinant hFH. One mouse dosed with recombinant mFH (**Figure 8C**) presented a less florid picture. Overall, the appearance of anti-FH antibodies in these kidney sections is consistent with the presence of anti-FH antibodies detected in plasma at levels that vary between individual animals but are higher in mice receiving recombinant FH.

## Discussion

We used PDI co-expression to boost FH production in yeast up to 100-fold. This could enable trials of therapeutic recombinant FH that, to date, have been hindered by a protein shortage. Inhibition of fluid-phase C3 consumption, suppression on self-surfaces of C3b amplification and contribution to iC3b and C3d(g) production are canonical functions of FH (32, 66). FH also sequesters by-products of lipid oxidation, is a ligand for CD11b and recognizes and promotes non-inflammatory clearance of injured cells (37). Which of its functions is impaired by a given disease-linked *CFH* mutation is often unclear. Consequently, therapeutic administration of full-length FH, lacking at-risk SNPs and hence possessing a full quota of functional sites, is worth exploring for selected, genotyped patients. Since the recipients would be heterozygous for *CFH*, there would probably be two allotypic variants of FH, as well as a set of FH-related proteins, in their circulation. Hence it seems unlikely there would be any previously unencountered epitopes present on the supplemental FH (provided it was appropriately glycosylated). Moreover, administration of full-length glycoprotein avoids concerns re the use of smaller versions of FH containing just some of its 20 domains (67–70). Moreover, FH could be more therapeutically effective than antibodies against soluble complement proteins, which generally lack specificity for self-surfaces.

We chose *P. pastoris* (71) because it grows in simple media and has been used to produce numerous recombinant proteins. Its use avoids oncogenic or viral nucleic acid as may occur when mammalian cells are utilised, and toxic cell-wall pyrogens such as generated by *E. coli.* We initially inserted a codon-modified gene for mFH under the *AOX1* promoter (72) in *P. pastoris* KM71H with a *S. cerevisiae* α-mating factor pre-pro peptide signal leader. This approach succeeded previously for hFH (52) although yields were below expected. In current work, the barely detectable amounts of mFH produced by *P. pastoris* in initial trials - not increased by selecting for strains with higher gene-copy numbers - suggested a production bottleneck. We noted reports of using *P. pastoris* to make disulfide-containing extracellular proteins, although none contain as many disulfides and domains (40 and 20 respectively) as FH.

Nascent polypeptides destined for secretion translocate to the ER where redox potential favours disulfides. Within the ER, PDI (73, 74) accelerates exchange between oxidized and reduced cysteine allowing the folding process to more quickly find the lowest-energy disulfide pattern normally coincident with the native one. Correctly folded proteins are transferred to the Golgi prior to secretion, but insufficiency of PDI could allow persistence within the ER of “mis-folded” conformations with non-native disulfides. These trigger quality-control pathways of the unfolded protein response (UPR) including elevated chaperonin production, translation arrest and ER-associated protein degradation (75, 76). Since the need to form 40 disulfides in overproduced FH could overwhelm the native capacity of yeast ER to promote correct disulfide formation, we chose to overproduce PDI alongside FH. So that PDI and FH production coincide we used the same *AOX1* promoter for both. The codon-modified *PDI* gene avoided extensive sequence identity between insert and chromosome. We incorporated *PDI* and *CFH* genes into the yeast genome sequentially. This created a versatile *PDI* ^+^ strain that could be transformed with heterologous genes other than *CFH*. The use here of separate plasmids with different selectable markers allowed various copy numbers to independently arise in different clones. Thus, we could produce and test multiple clones and identify empirically best producers.

Several reports describe co-production of proteins with chaperonins to increase yields (77–87), but our production gains were unprecedented. In no reported case was the target protein a multiple-domain disulfide-rich protein. Even one misfolded domain out of the 20 in FH could trigger the UPR. It follows that the chances of a many-domain protein passing quality checks increase exponentially if each domain has just a slightly higher likelihood of achieving its native disulfide-bonding pattern. Thus, the more disulfide-containing domains in a protein, the greater the productivity gains to expect from PDI overproduction. Encouragingly, this implies other multiple-domain disulfide-rich proteins - even those that are not produced in detectable quantities by a standard *P. pastoris* strain - could be produced using our approach.

Tested *in vitro*, recombinant *P. pastoris* mFH bound mC3b, and decayed mC3bBb, as effectively as native mFH despite a lack of N-glycans and the presence of clipped material in our sample, not easily removed. Following IP injection of our recombinant mFH into *CFH* ^-/-^ mice, plasma FH levels were elevated at six hours but dropped back by 24 hours. Conversely, as expected (45), FH remained elevated in plasma of *CFH^-/-^* mice 24 hours post-injection of native (mouse serum-derived) FH and then declined slowly. Rapid clearance of recombinant FH was previously observed for full-length hFH produced in moss with partially humanized N-glycans (51). A short half-life was likewise observed for CHO cell-produced “mini” hFH (missing modules 6-17) (69). Both were virtually undetectable after six hours. Full-length FH is too large for glomerular filtration so given the dearth at this time point of anti-FH antibodies and a lack of receptors for single GlcNAc residues, recombinant FH is likely sequestered within extracellular matrix (ECM) including that of the GBM. It was previously shown that CHO cell-produced fluorescently tagged recombinant mFH was detected in GBM shortly after injection into *CFH* ^-/-^ mice (41). Native hFH carries disialylated N-glycans (88) and also has polyanion-binding sites in domains 7 and 20 (62). A lack of negatively charged N-glycans on our recombinant FH is one explanation for an increased avidity for ECM and hence more rapid loss from plasma. This could simply be an electrostatic effect. Another theory, predicated on the postulated existence of *intra*molecular contacts between FH-borne sialic acids and sialic acid-binding sites on FH, proposes that when interacting with recombinant FH, GBM sialic acids would not have to compete with sialic acids covalently attached to FH (62).

Elevated recombinant mFH in plasma was unsustainable even with ten daily injections. A similar observation was made (29) following multiple daily injections of native hFH. By day five, levels of our recombinant FH were 50% of WT, insufficient to arrest a declining trend in C3 levels. Further deterioration of FH (and C3) levels between five and eleven days is likely connected to emergence of anti-FH antibodies. *CFH* ^-/-^ mice should have some immune tolerance to mFH due to their mFH-related proteins (mFHR) A, B and C (89), and despite the FH knockout altering immune tolerance (90). Indeed, **Figure 6** shows that a substantial antibody titre arose between days 5 and 11 only in the case of recombinant FH. Anti-FH antibodies induced in WT mice by recombinant mFH, recognized recombinant FH selectively (**Figure 7A**), suggesting that epitopes might include GlcNAc residues or the “frayed ends” of the clipped backbone. A separate 41-day experiment (with FH injected at days 0 and 24) confirmed recombinant mFH is more immunogenic than native mFH in *CFH* ^-/-^ mice (**Figure 7B**).

Administration of recombinant versions of mFH and hFH temporarily raised plasma levels of intact C3 (**Figure 6**). Dose for dose, however, they resulted in smaller and shorter-lived elevations of intact C3 than did native mFH. No C3-staining in mesangial or interstitial tubular regions was detected in recombinant mFH-treated mice consistent with sub-wild-type levels of C3 in plasma. These observations may be explained as secondary consequences of the more rapid clearance of recombinant FH, given that plasma levels of intact C3 mirrored those of FH.

In previous multiple daily-dose experiments, using native hFH, glomerular staining with polyclonal anti-C3 antibody declined after five days but reappeared after ten (29). This breakdown in the propensity of daily hFH injections to control C3 fragment deposition between five and ten days was linked to the appearance of anti-hFH antibodies. In the current study, treatment with recombinant mFH likewise significantly reduced glomerular anti-C3 staining (**Figure 6**). But, unlike in the previous study, this reduction persisted beyond the tenth daily injection of recombinant mFH, despite low FH and C3 levels in plasma.

Plasma levels of FH do not necessarily reflect how much FH is present in the GBM where FH can bind many partners including GAGs, sialic acids (see above) and, if present, C3 fragments. Thus, recombinant FH could have a long residency time. When FH is continuously supplied by regular IP injections, and notwithstanding an increasing titre of circulating anti-FH antibodies, a steady-state quantity of functional FH could be established in the GBM that is adequate for preventing both further local generation and deposition of nascent C3b and proteolytic processing of existing C3b. Indeed, in both previous and current studies, staining for C3d(g) was also reduced, compared to controls, after ten days of recombinant mFH treatment.

A serendipitous scenario, in which circulating recombinant FH and C3 levels are low while recombinant FH residency in the GBM is high, could be optimal from the narrow perspective of resolving C3 fragment deposition in glomeruli. It is reminiscent of the *CFH^-/-^/Crry^-/-^* double-knockout mouse in which a mild phenotype (compared to *CFH* ^-/-^) was attributed to plasma C3 depletion. It is the inverse of the scenario underlying the severe phenotype associated with homozygosity for the mFH mutant W1206R (42). In these animals fluid-phase AP regulation and hence C3 levels are maintained but C3b amplification on surfaces is unregulated because W1183R has low affinity for surfaces. The benefit observed in our study might be temporary as, longer term, higher titres develop of anti-FH antibodies or antibodies arising to the GBM-bound form of FH, and antibody-antigen complexes could accumulate in the kidney. It is highly unlikely that human FH produced in this strain would be suitable for human therapy. However, the boost in yield obtained by co-expression with *PDI* suggest that migration of this strategy to a glycoengineered strain of *P. pastoris* (91) capable of decorating proteins with complex, mammalian-like N-glycans would be worth investigating.

In conclusion we have developed a platform for production of large quantities of the large and important category of proteins that are rich in disulfide bonds and possess many domains. These are currently heavily under-represented in the list of recombinant proteins. Our recombinant versions of FH are biologically active *in vitro* and *in vivo*. An obvious way to reduce the immunogenicity of our products would be to circumvent “clipping” and to use a strain of *P. pastoris* that decorates glycoproteins with the desired N-glycans to yield FH with a native conformation and immune tolerance. Serendipitously we show that a good, but not necessarily clinically desirable, means of resolving C3 fragment deposition in glomeruli is to leave C3 consumption in fluid phase largely unchecked while targeting regulation to the GBM.

## Data Availability Statement

The original contributions presented in the study are included in the article/**Supplementary Material**. Further inquiries can be directed to the corresponding author.

## Ethics Statement

All mice were housed in specific pathogen-free conditions; procedures were performed according to institutional guidelines and approved by the United Kingdom Home Office.

## Author Contributions

HK designed and performed experiments, interpreted data, and helped write the paper. AH contributed to experimental design and supplied materials. EM designed and performed experiments. DA designed, performed, and interpreted experiments. TM and HL-B designed experiments and performed measurements. YY helped designed experiments and performed measurements. IP and HD performed measurements. AR helped to conceive the project and design experiments. KM designed experiments, supplied animals, and helped interpret results and write the paper. MP helped to conceive the project, supplied animals, and helped interpret results and write the paper. PB helped to conceive the project, interpreted results, and wrote the initial draft of the paper. All authors contributed to the article and approved the submitted version.

## Funding

HK was an MRC (G1001971) Clinical Training Fellow. MP is a Wellcome Trust Senior Fellow in Clinical Science (212252/Z/18/Z). KM was funded by the Northern Counties Kidney Research Fund and Kidney Research UK project grants (RP7/2015 & RP_006_20170301). DA is funded by IBioIC (2019-1-6/2017-166B). AR was a Wellcome Trust Intermediate Clinical Fellow (WT085226; 2009-2014).

## Conflict of Interest

KM has received consultancy or research income from Gemini Therapeutics Inc, Freeline Therapeutics, MPM Capital, Idorsia Pharmaceuticals Ltd and Catalyst Biosciences. PB is a scientific co-founder of, and has received consultancy and research income from, Gemini Therapeutics Inc. AH is founder and Chief Scientific Officer of Invizius. EM is currently an employee of Invizius. AR has been employed at GlaxoSmithKline since October 2014. Results reported in this work were undertaken during her Wellcome Trust Intermediate Clinical Fellowship. Her spouse, David Kavanagh, is head of the National Renal Complement Therapeutics Centre, UK, and a board member and scientific advisor to Gyroscope Therapeutics Ltd. PNB and AR are co-authors on a recombinant CFH patent application (EP10803266A).

The remaining authors declare that the research was conducted in the absence of any commercial or financial relationships that could be construed as a potential conflict of interest.
