# Supplementary material for: Murine Factor H Co-Produced in Yeast With Protein Disulfide Isomerase Ameliorated C3 Dysregulation in Factor H-Deficient Mice
Source: Front Immunol. 2021 May 12;12:681098. doi: 10.3389/fimmu.2021.681098 (PMC8149785; doi:10.3389/fimmu.2021.681098)
Supplement: Supplementary file 1 [file DataSheet_1.pdf]

Murine factor H co-produced in yeast with protein disulfide isomerase ameliorated C3 dysregulation in Factor H-deficient mice

Heather Kerr, Andrew P Herbert, Elisavet Makou, Dariusz Abramczyk, Talat H Malik, Hannah Lomax-Browne, Yi Yang, Isabel Y Pappworth, Harriet Denton, Anna Richards, Kevin J Marchbank, Matthew C Pickering and Paul N Barlow

SUPPLEMENTARY DATA

Supplementary Figure 1 *The sequences of synthetic DNA molecules*
Supplementary Figure 2 *Production and purification of recombinant murine factor H*
Supplementary Figure 3 *qPCR and RT-PCR*
Supplementary Figure 4 *Factor I cofactor assay*

A: Synthetic DNA coding for mouse complement factor H

GAAGATTGTAAAGGTCCTCCGCCTAGAGAGAACTCGGAAATCTTGTCAGGTTCCTGGTCTGAGCAACTCTATCCAGAGGGTAC
GCAAGCTACCTACAAATGCCGTCCAGGTTATAGAACACTTGGAACCATCGTCAAGGTTTGTAAAAACGGAAAATGGGTTGCCT
CAAATCCCTCCAGAATTTGTCGTAAAAAGCCATGTGGTCATCCGGGTGATACACCTTTCGGTTCCTTCAGATTGGCAGTCGGT
TCCCAATTTGAATTTGGTGCAAAAGTCGTGTACACTTGTGATGATGGTTATCAGCTGTTGGGTGAAATTGACTACAGGGAATG
TGGTGCTGATGGTTGGATCAATGACATTCCATTGTGTGAAGTCGTGAAGTGTCTACCAGTTACTGAATTGGAAAATGGCAGAA
TTGTTTCCGGAGCAGCCGAAACCGATCAGGAGTACTACTTCGGTCAAGTTGTTCGTTTCGAGTGTAATTCTGGCTTCAAGATT
GAGGGACATAAGGAGATCCACTGTTCCGAAAATGGTTTGTGGAGCAATGAGAAACCAAGATGTGTGGAGATTTTGTGTACTCC
TCCTAGAGTCGAGAATGGCGACGGTATAAACGTAAAACCTGTTTACAAGGAAAACGAAAGATACCATTACAAGTGCAAGCACG
GTTATGTGCCTAAGGAAAGGGGTGATGCTGTCTGCACAGGCTCAGGTTGGAGCAGCCAGCCTTTTTGCGAGGAAAAGAGATGT
AGCCCACCATATATCTTAAACGGAATCTACACCCCACATAGGATCATCCACCGTTCGGACGATGAAATCCGTTATGAATGTAA
CTACGGCTTCTACCCTGTTACAGGTTCAACTGTCAGTAAGTGTACGCCAACTGGTTGGATTCCTGTTCCAAGATGTACACTTA
AACCTTGTGAATTTCCACAATTCAAGTATGGCAGGTTGTACTATGAAGAGTCATTACGTCCGAATTTCCCAGTCTCGATCGGC
AACAAGTACAGCTACAAGTGCGATAATGGATTCTCTCCTCCATCAGGATACAGCTGGGACTATCTGCGTTGTACGGCTCAGGG
TTGGGAACCAGAAGTACCTTGTGTGAGAAAATGCGTTTTTCACTACGTGGAGAATGGAGATTCCGCTTACTGGGAGAAAGTGT
ACGTTCAAGGTCAGTCACTTAAGGTTCAATGTTACAATGGTTACTCTCTTCAAAACGGTCAAGATACTATGACATGTACTGAG
AACGGATGGTCGCCTCCACCTAAGTGTATCAGAATCAAAACTTGCTCGGCTTCAGACATTCACATTGATAATGGTTTCCTGTC
AGAGTCATCAAGTATCTACGCTTTGAATAGAGAAACTAGTTACAGATGCAAACAGGGTTACGTTACAAACACCGGAGAAATTA
GTGGTTCCATCACATGTTTGCAAAATGGATGGAGCCCACAGCCCTCTTGCATTAAGTCCTGTGATATGCCTGTGTTTGAAAAC
TCAATTACAAAGAACACAAGGACCTGGTTCAAACTTAACGATAAGTTAGACTATGAGTGCTTGGTGGGTTTTGAGAATGAGTA
TAAGCACACTAAAGGATCAATTACTTGTACTTATTACGGTTGGTCTGACACTCCTTCGTGTTACGAAAGAGAGTGTTCCGTCC
CAACTTTGGACAGGAAGTTGGTTGTTTCCCCTAGAAAGGAGAAGTACCGTGTGGGTGACTTATTGGAGTTCTCATGCCATTCG
GGTCACAGAGTGGGTCCTGATTCTGTTCAGTGTTATCACTTTGGTTGGTCACCAGGTTTTCCTACGTGTAAGGGTCAAGTGGC
AAGCTGCGCACCACCTCTGGAGATTCTAAACGGAGAAATCAACGGAGCCAAAAAGGTTGAGTATTCACATGGTGAAGTGGTCA
AATATGATTGCAAGCCAAGATTTCTGTTGAAAGGACCTAACAAAATACAATGTGTTGACGGTAATTGGACAACTCTGCCAGTG
TGTATCGAGGAGGAGCGTACATGTGGTGACATCCCAGAGCTAGAGCATGGAAGTGCTAAGTGCAGTGTGCCACCTTACCATCA
CGGAGACTCGGTCGAGTTCATCTGTGAGGAGAATTTTACTATGATTGGTCATGGTTCTGTCTCTTGTATCAGTGGCAAATGGA
CCCAATTGCCAAAATGCGTGGCAACTGATCAACTGGAAAAATGCCGTGTTCTGAAATCCACGGGTATTGAGGCTATTAAACCC
AAATTGACTGAGTTTACACATAATTCTACGATGGACTACAAGTGTCGAGATAAGCAGGAATATGAACGTTCTATTTGTATTAA
TGGTAAGTGGGACCCAGAGCCAAACTGTACCTCCAAAACTTCGTGCCCACCACCTCCTCAAATTCCAAACACTCAAGTTATAG
AAACAACCGTCAAGTACTTAGACGGTGAAAAGCTCTCCGTCTTATGTCAAGATAATTACCTAACTCAAGATTCCGAGGAAATG
GTGTGTAAGGATGGACGTTGGCAATCACTGCCTAGATGTATTGAGAAAATCCCTTGTTCCCAGCCACCTACAATAGAACACGG
TTCCATAAACCTGCCAAGATCGTCTGAGGAAAGGCGTGATTCCATTGAATCTAGTAGCCATGAACATGGTACAACCTTCTCCT
ACGTCTGCGACGACGGATTCAGAATCCCAGAGGAAAATAGAATTACGTGTTATATGGGAAAATGGTCCACCCCTCCTCGTTGC
GTTGGTCTGCCATGCGGACCACCTCCATCGATTCCCTTGGGTACTGTGAGTTTGGAGCTTGAAAGCTACCAGCACGGAGAAGA
GGTTACTTATCACTGCTCCACTGGATTTGGTATTGACGGTCCAGCTTTCATCATCTGCGAGGGAGGTAAGTGGAGCGACCCTC
CAAAGTGCATCAAGACAGACTGCGACGTGTTACCTACTGTCAAAAACGCAATCATAAGGGGTAAAAGCAAAAAGTCATACAGA
ACTGGTGAACAGGTTACTTTCAGATGCCAGAGCCCATACCAAATGAATGGTAGCGATACAGTTACTTGTGTTAACAGCAGATG
GATCGGTCAGCCAGTTTGTAAAGATAATTCATGTGTGGACCCACCCCATGTTCCGAATGCAACAATCGTTACCAGGACGAAAA
ACAAGTATTTGCATGGAGATAGAGTAAGATACGAATGTAATAAGCCATTGGAACTTTTTGGCCAAGTGGAAGTTATGTGCGAG
AATGGTATTTGGACGGAAAAGCCAAAATGTAGAGATTCCACTGGAAAGTGTGGACCTCCACCACCCATTGATAACGGTGACAT
TACATCCCTGTCCCTGCCAGTTTATGAGCCTTTGTCGTCTGTTGAATACCAGTGTCAAAAATACTATTTGCTGAAAGGTAAGA
AAACAATTACATGTACTAATGGCAAGTGGTCCGAGCCACCAACTTGCCTGCACGCTTGTGTTATTCCAGAGAATATCATGGAA
TCCCATAACATTATTCTCAAGTGGAGGCACACAGAGAAGATATACAGCCATTCCGGTGAGGACATCGAGTTTGGTTGTAAGTA
TGGATATTACAAAGCTAGAGACTCACCACCATTCAGGACAAAGTGTATAAACGGTACTATCAACTACCCAACTTGT GTTTAG

B: Synthetic DNA coding for P. pastoris PDI

GGATCCAAAACGATGCAGTTCAACTGGGACATCAAGACTGTTGCTTCCATCTTGTCCGCTTTGACTTTGGCTCAAGCTTCTGACCAAGAGGCT
ATTGCTCCAGAAGATTCCCACGTTGTTAAGTTGACTGAGGCTACTTTCGAGTCCTTCATCACTTCCAACCCACACGTTTTGGCTGAGTTTTTC
GCTCCATGGTGTGGTCACTGTAAGAAGTTGGGTCCAGAATTGGTTTCCGCTGCTGAGATTTTGAAGGACAACGAGCAGGTTAAGATCGCTCAG
ATCGACTGTACTGAAGAGAAAGAGTTGTGTCAGGGTTACGAGATCAAGGGTTACCCAACTTTGAAGGTTTTCCACGGTGAGGTTGAAGTTCCA
TCCGACTACCAAGGTCAAAGACAATCCCAATCCATCGTTTCCTACATGTTGAAGCAGTCCTTGCCACCAGTTTCCGAGATCAACGCTACTAAG
GATTTGGACGACACTATCGCTGAGGCTAAAGAGCCAGTTATCGTTCAGGTTTTGCCAGAGGACGCTTCTAACTTGGAGTCCAACACTACTTTC
TACGGTGTTGCTGGTACTTTGAGAGAGAAGTTCACTTTCGTTTCCACTAAGTCCACTGACTACGCTAAGAAGTACACTTCCGACTCCACTCCA
GCTTACTTGTTGGTTAGACCAGGTGAGGAACCATCCGTTTACTCTGGTGAAGAATTGGACGAGACTCATTTGGTTCACTGGATCGACATTGAG
TCCAAGCCTTTGTTCGGTGACATTGACGGTTCCACTTTCAAGTCCTACGCTGAAGCTAACATCCCATTGGCTTACTACTTCTACGAGAACGAG
GAACAGAGAGCCGCTGCTGCTGACATTATTAAGCCATTCGCTAAAGAACAAAGAGGTAAGATCAACTTCGTTGGTTTGGACGCTGTTAAGTTC
GGTAAGCACGCTAAAAACTTGAACATGGACGAAGAGAAGTTGCCTTTGTTTGTTATCCACGACTTGGTTTCCAACAAGAAGTTCGGAGTTCCA
CAGGACCAAGAGTTGACTAACAAGGACGTTACTGAGTTGATCGAGAAGTTTATCGCTGGTGAGGCTGAGCCAATCGTTAAGTCTGAACCTATC
CCAGAGATCCAAGAGGAAAAGGTTTTCAAGTTGGTTGGTAAGGCTCACGACGAGGTTGTTTTCGACGAATCTAAGGACGTTTTGGTTAAGTAC
TACGCTCCTTGGTGTGGACACTGTAAAAGAATGGCTCCAGCTTATGAAGAGTTGGCTACTTTGTACGCTAACGACGAAGATGCTTCCTCCAAG
GTTGTTATCGCTAAGTTGGACCACACTTTGAACGACGTTGATAACGTTGACATCCAGGGATACCCTACATTGATCTTGTACCCAGCTGGTGAC
AAGTCCAACCCTCAGTTGTACGATGGTTCTAGAGACTTGGAATCCTTGGCTGAATTCGTTAAGGAAAGAGGTACTCACAAGGTTGACGCTTTG
GCTTTGAGACCTGTTGAGGAAGAAAAAGAGGCTGAAGAGGAAGCTGAATCTGAAGCTGATGCTCACGATGAGTTGTAAGCGGCCGC

Supplementary Figure 1 *The sequences of synthetic DNA*
(A) This sequence coding for murine factor H was codon-optimised and supplied by DNA 2.0 (now ATUM). ***(B)*** This sequence coding for *P. pastoris* protein disulfide isomerase was codon-optimised and supplied by GeneArt.

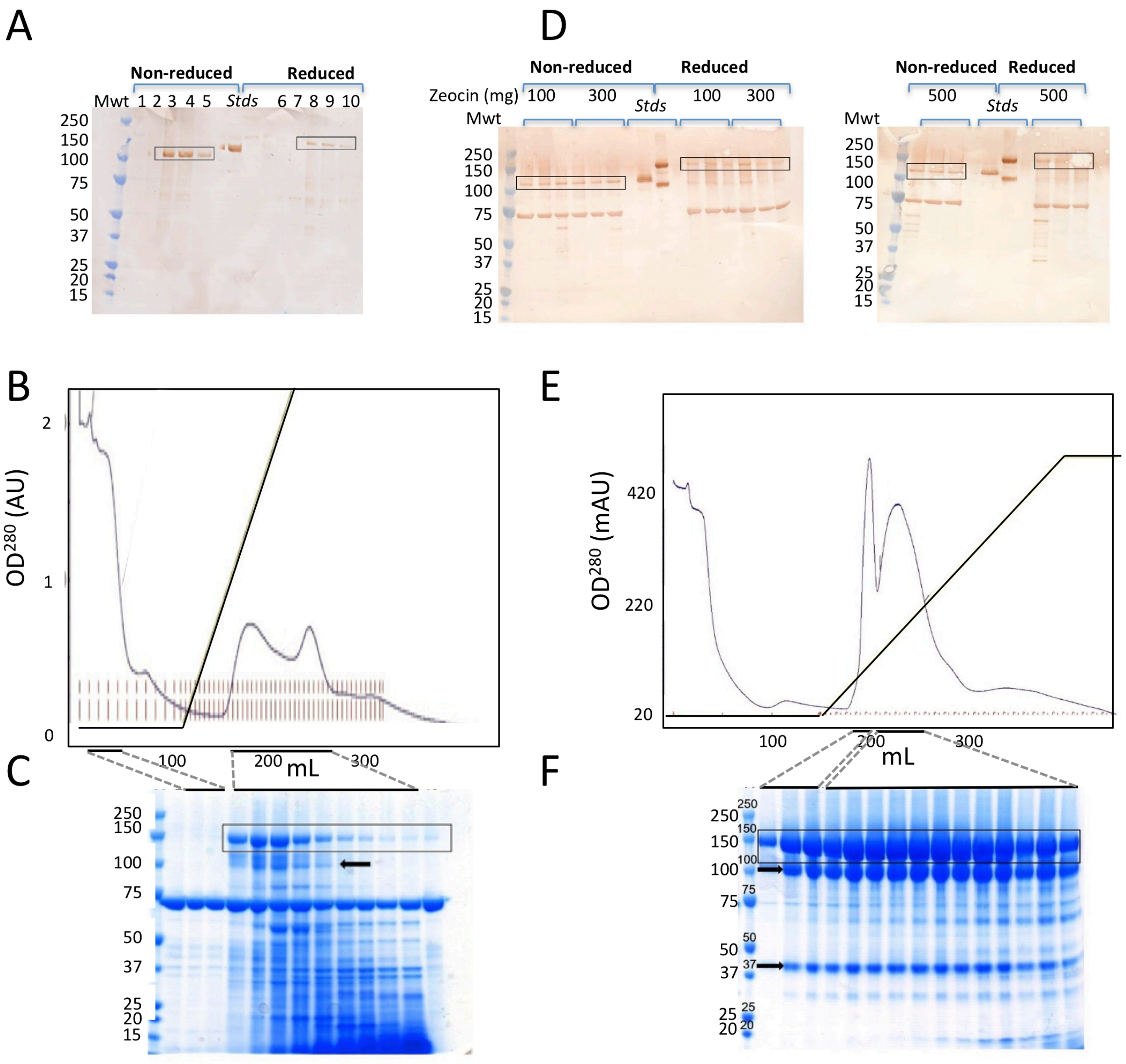

Supplementary Figure 2 *Production and purification of recombinant murine factor H*
(A) Very low quantities of target protein were produced by *P. pastoris* (KM71H) cells transformed with synthetic genes for mFH. Detection of mFH by SDS-PAGE and western blot (WB) required prior capture on heparin-affinity resin and high-salt elution. Shown is the outcome of running Endo H_f-treated 10-µL aliquots from five sequential 1-mL fractions under non-reducing or reducing conditions, with mouse serum as a standard (*Std*). Primary Ab: goat anti-hFH; secondary Ab rabbit anti-goat IgG-HRP. No candidate band for mFH could be distinguished after Coomassie-Blue staining (not shown). ***(B)*** Fermentation of KM71H confirmed FH production, but at low yield. From a 10-L fermentation, diluted supernatant was adjusted to pH 6 before loading on a 75-mL SP-Sepharose column, then eluting with 0.05 M – 1.0 M NaCl over four column volumes to generate the chromatogram shown. ***(C)*** Results (Coomassie Blue staining) of SDS-PAGE performed (under reducing conditions) on 10-µl aliquots from the indicated 2-mL fractions. Black boxes highlight bands corresponding to 155-KDa mFH. The arrow indicates evidence, under reducing conditions only, for partial proteolysis. The ~75 kDa band is Endo H_f. ***(D)*** In contrast to KM71H, KM71-PDI produces sufficient mFH in small-scale trials to be detected without enrichment by WB. Positive transformants were selected on agar plates using various zeocin concentrations as shown. Three single

colonies from each plate were used to inoculate 25-mL cultures. Each lane was loaded with 10 μL of Endo H_f-treated supernatant. Samples were run (on two gels), under reducing or non-reducing conditions. Std = previously prepared sample of recombinant mFH. Primary Ab, sheep anti-hFH; secondary Ab was rabbit anti-sheep IgG. Cross-reactivity with Endo H_f (at 75 kD) is evident. ***(E)*** Large quantities of mFH are produced in a 10-L fermentor. 6 L of supernatant from the cell culture was loaded onto a Toyopearl 650 column. Shown is the elution profile obtained with a 0.05 - 1 M NaCl gradient applied over four column volumes. ***(F)*** For SDS-PAGE, 10 μL from each indicated fraction was loaded. The band corresponding to mFH is boxed. The arrow indicates “clipped” protein (the band at ~75 kDa is Endo H_f).

A

Primer ID	Sequence 5'-3'	Amplicon (bp)/purpose
**mFH-FvQ2-215**	GCAGCCGAAACCGATCAGGAG	100 bp / qPCR recombinant mFH
**mFH-RvQ2-212**	CCGTCGCCATTCTCGACTC	
**mFH-FvQ1-213**	GTGGGACCCAGAGCCAAACTG	137 bp / qPCR and qRT-PCR recombinant mFH
**mFH-RvQ1-214**	GACGGAGAGCTTTTCACCGTC	
**PDI-F-23**	CTGTTGCTTCCATCTTGTCCGC	132bp/ qPCR recombinant PDI
**PDI-RQ2-257**	CGTGTGGGTTGGAAGTGATGAAG	
**PDI-F-ver2-Q1-24**	GCCAGAGGACGCTTCTAACTTGG	135bp/ qPCR and qRT-PCR recombinant PDI
**PDI-R-ver1-Q1-25**	GCTGGAGTGGAGTCGGAAGTG	
**PDH-PDA1-FQ2-258**	GCGAAGCCGTTCTTCTCGAAG	133bp/ qPCR housekeeping gene PDH-PDA
**PDH-PDA1-RQ2-259**	CTGAACTCGCTCCTCCCCC	
**PDH-PDA1 FQ1-149**	CTGGACTTCCTTGACAGAGGC	118bp/ qPCR housekeeping gene PDH-PDA
**PDH-PDA1 RQ1-150**	CCAAGAAGCCGTGGCTGTAG	
**FQ1-ACT1-35**	TTCGTCGGTGACGAGGCTC	137bp/ qPCR and qRT-PCR housekeeping gene ACT1
**RQ1-ACT1-36**	GGGGCCAGACGCAACTCG	

B

Strain	Gene Copy Number	
	mFH	PDI
**KM71-*PDI-mFH* (Mut^S)**	1.99±0.2	1.8±0.1

C

Strain	Expression Level	
	PDI	*mFH*
**KM71-*PDI* (Mut^S)**	285 ±1	N/A
KM71-*PDI-mFH* **(Mut^S)**	205 ±10	98 ±13

Supplementary Figure 3 *Gene copy number and transcript abundance*

(A) The primers (Integrated DNA Technologies) used for qPCR and qRT-PCR. Validation of primers showed an efficiency of between 98% and 103%, and slope values in the range of -3.24 to -3.35. Each gene (with the exception of ACT1) for qPCR was targeted by two pairs of primers corresponding to two distant locations within the gene. ***(B)*** An estimation of gene copy numbers in the strain co-expressing *PDI* and *mFH*. Extraction of genomic DNA (gDNA) of *P. pastoris* was performed using the protocol provided for the MasterPure Yeast DNA Purification Kit (Epicentre) modified to include an additional RNAse A treatment and an extra purification step using the Zymo Spin III column clean-up system (Zymo-Research). The purity of gDNA was calculated using the Qubit dsDNA BR Assay Kit (Thermo Fisher). The qPCR reactions were performed using the Luna Universal qPCR Master Mix protocol (New England Biolabs) using reaction volumes of 10 µL in 96-well plates adjusted for the LightCycler 96 system (Roche).The cycle threshold (C_T) values were obtained for two biological repeats with three technical duplicates and calculated by software LightCycler480. Gene copy numbers were calculated by normalizing these separately with respect to the two housekeeping genes, *ACT1* and *PDH-PDA*, assumed to be present as single copies. ***(C)*** *PDI* and *mFH* expression levels post-induction compared to the expression level of *ACT1*. The growth and induction of cells is described in the Methods section. Harvested cells were disrupted with glass beads in the presence of Tri-Reagent (Zymo Research) using the PreCellys cell disruptor (Bertin Instruments). The resulting cell lysates were used for RNA extraction (using the Tri-Reagent standard protocol). Samples of RNA Isolated by isopropanol precipitation were dissolved in 10 mM Tris, pH 8.0, and treated with DNAse I (RNAase free) (Thermo Fisher). Following enzyme inactivation, RNA was purified using the Zymo-Spin IIICG column clean-up system (Zymo Research) and its purity was estimated using the Qubit RNA BR Assay Kit (Thermo Fisher). DNAse-free total RNA was used as a template in a SuperScript IV Reverse Transcriptase (Invitrogen) reaction following the manufacturer's protocol (and using random hexamers in the reaction) to generate cDNA. cDNA in tenfold dilutions was used in qPCR reactions following the protocol for Luna Universal qPCR Master Mix (New England Biolabs). Samples were analysed using the LightCycler 96System (Roche) in two biological repeats with three technical duplicates. The levels of *mFH* and *PDI* transcript was normalized against the level of *ACT1* transcript via the $2{-}\Delta\Delta C_T$ method. After 40 cycles the specificity of the amplicons was confirmed *via* a melting curve.

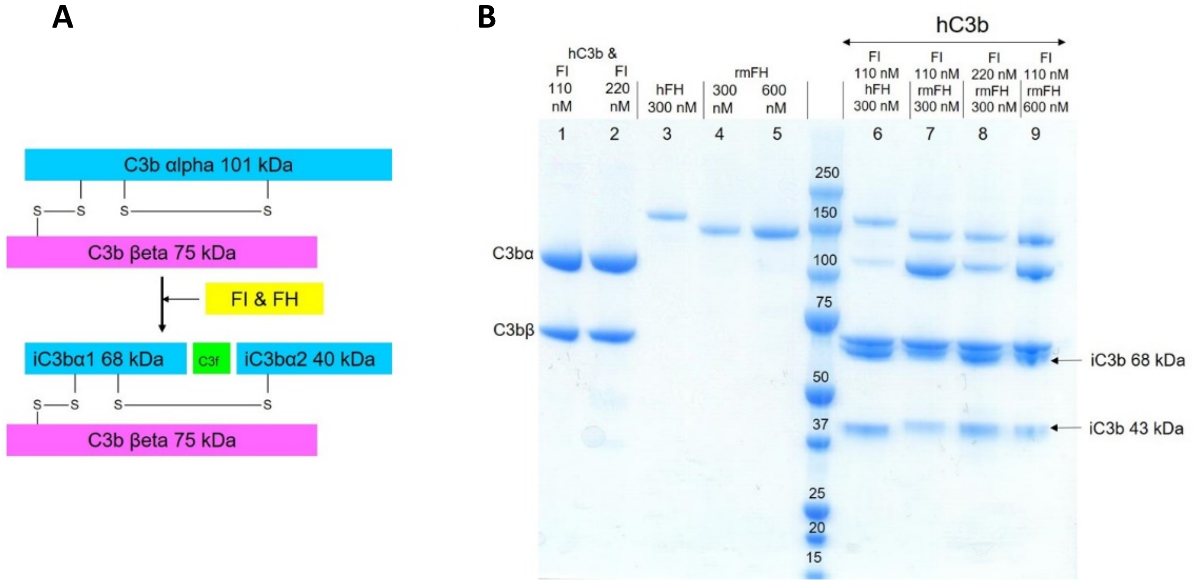

Supplementary Figure 4 *Factor I cofactor assay*

(A) Schematic illustrating C3b α and β chains with disulfide bonds, and cleavage at two positions of the α'-chain by factor I (FI) in the presence of factor H (FH) to generate iC3b and C3f. (B) Lanes 1 and 2: no-FH samples containing 850 nM human C3b and the stated amount of human FI. Lanes 3-5: no-FI, no-C3b samples containing human FH (hFH) or recombinant mouse FH (rmFH) at the stated concentrations. Lanes 6-9: Samples contained 850 nM human C3b and the stated concentrations of human FI and either hFH or recombinant mFH. Note that the recombinant mFH used in this assay had undergone an extra purification step on ceramic hydroxyapatite.
